# Text-Book of Diseases of Women

**Published:** 1900-10

**Authors:** 


TEXT-BOOK OF DISEASES OF WOMEN.—By Chas. B. Penrose, M. D., Ph. D., Professor of Gynecology in the University of Pennsylvania, etc., Philadelphia. Illustrated. Third edition. Revised. 531 pages. Price: Cloth, 3.75. Philadelphia: W. B. Saunders, 925 Walnut Street. 1900.

Penrose is connected with one of the greatest schools in this country, and is a standard authority of the subject of diseases of women. His vast experience and careful methods have qualified him to write a book, and he wrote it several years ago. The volume under consideration is the third edition. It has been thoroughly revised and brought up to date. The author says that the book is intended for the medical student, not meaning alone the young man in college, but the practitioner who studies and tries to keep up with the advancements in his profession.

The work is happily illustrated, and conveniently arranged. In every respect it is a standard and first-class work. T. J. B.

